# A targeted capture approach to generating reference sequence databases for chloroplast gene regions

**DOI:** 10.1002/ece3.8816

**Published:** 2022-04-11

**Authors:** Nicole R. Foster, Kor‐jent van Dijk, Ed Biffin, Jennifer M. Young, Vicki A. Thomson, Bronwyn M. Gillanders, Alice R. Jones, Michelle Waycott

**Affiliations:** ^1^ 1066 School of Biological Sciences University of Adelaide Adelaide South Australia Australia; ^2^ State Herbarium of South Australia Botanic Gardens and State Herbarium Adelaide South Australia Australia; ^3^ 1065 College of Science and Engineering Flinders University South Australia Australia

**Keywords:** angiosperms, barcoding, hybridization capture, plastid

## Abstract

Metabarcoding has improved the way we understand plants within our environment, from their ecology and conservation to invasive species management. The notion of identifying plant taxa within environmental samples relies on the ability to match unknown sequences to known reference libraries. Without comprehensive reference databases, species can go undetected or be incorrectly assigned, leading to false‐positive and false‐negative detections. To improve our ability to generate reference sequence databases, we developed a targeted capture approach using the OZBaits_CP V1.0 set, designed to capture chloroplast gene regions across the entirety of flowering plant diversity. We focused on generating a reference database for coastal temperate plant species given the lack of reference sequences for these taxa. Our approach was successful across all specimens with a target gene recovery rate of 92%, which was achieved in a single assay (i.e., samples were pooled), thus making this approach much faster and more efficient than standard barcoding. Further testing of this database highlighted 80% of all samples could be discriminated to family level across all gene regions with some genes achieving greater resolution than others—which was also dependent on the taxon of interest. Thus, we demonstrate the importance of generating reference sequences across multiple chloroplast gene regions as no single loci are sufficient to discriminate across all plant groups. The targeted capture approach outlined in this study provides a way forward to achieve this.

## Graphical abstract

This study has applied a targeted capture approach to generate a DNA reference sequence database for coastal temperate flora. We implemented a bait set designed to capture 20 chloroplast gene regions in a single assay, thus highlighting an approach for efficient generation of plant reference databases across multiple gene regions.

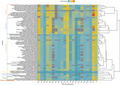

## 1 INTRODUCTION

The amplification of DNA sequences from environmental samples, termed metabarcoding, has been extensively applied to monitor invasive species, detect changes in communities over time, monitor animal diets and more (Deiner et al., 2017; Ruppert et al., 2019). For metabarcoding to be successful, DNA sequences recovered from environmental samples need to be accurately matched to sequences in online repositories/databases. Incomplete reference databases are a common limiting factor to plant metabarcoding studies (Dormontt et al., 2018), which is due to both the difficulty in generating plant barcodes and the lack of a universal, discriminatory gene region across all plant groups (Taberlet et al., 2012).

Generating standardized and comprehensive reference DNA sequence databases for plants is more challenging than it is for animals. The standard metabarcoding region for animal DNA is the mitochondrial cytochrome c oxidase subunit 1 (CO1) region (Liu et al., 2017); however, an equally informative region does not exist for plants (Dormontt et al., 2018). Plant mitochondria have a very low rate of nucleotide substitution (Hollingsworth et al., 2011), and can commonly undergo genome rearrangement, which makes them technically challenging and not a suitable barcoding region. Up to now, the organellar chloroplast genome regions *matK*, *rbcL*, and *trnH*‐*psbA* have been used as barcoding regions for plants (CBOL Plant Working Group, 2009), as well as the ribosomal nuclear region, the internal transcribed spacer (ITS) (Hollingsworth et al., 2016).

To improve reference sequence generation for plant species and ensure compatibility with metabarcoding research, we propose multiple chloroplast barcodes be generated in parallel for plant taxa. Conventional (PCR‐based) barcoding can be costly and time‐consuming as only a single region can be amplified per PCR (Jones et al., 2021). More recent approaches to generating chloroplast reference data include genome skimming (Straub et al., 2012), which does generate data for multiple gene regions, but this is not always of high quality, nor can multiple regions of interest be reliably recovered across all samples. Furthermore, this approach requires high sequencing effort, bioinformatic processing, and assembly, which can be challenging for chloroplast genomes (~150 KB). An alternative approach to generating chloroplast gene references is targeted or hybridization capture (Weitemier et al., 2014). This approach involves designing RNA “baits” that capture genetic regions of interest—in this case, chloroplast gene regions—and retain these while unwanted DNA is removed. Subsequent sequencing on next‐generation sequencing (NGS) platforms is efficient because the target regions are well represented in post‐capture libraries and multiple samples can be pooled within sequencing libraries.

This study implemented a targeted capture approach to reference generation using a bait set designed to capture across 20 chloroplast gene regions for all flowering plants. Thus, for a similar cost of generating references for the standard barcodes, *matK*, *rbcL*, and *trnH*‐*psbA*, 20 chloroplast gene region references could be generated instead. We tested this approach by creating a database of temperate coastal plants, given the availability of voucher specimens and the need for a reference database of temperate coastal plant taxa. We quantified the success of this approach by documenting the number of genetic regions recovered for each species and demonstrated the ability of this database to identify unknown sequences. Additionally, given the unique situation of having references for multiple chloroplast gene regions, we assessed the ability of these regions to separate taxa based on genetic distance both separately and when gene regions were combined iteratively.

## 2 METHODS

### 2.1 Generating the reference database

#### 2.1.1 Sample collection and DNA Extraction

A total of 93 coastal plant specimens were collated from a combination of field collections and previously collected herbarium specimens common across temperate Australian extant coastal communities. These specimens included key family groups from seagrass, saltmarsh, mangroves, and coastal plants (sample and location information can be found in Appendix S1: Table A1). Field collections were vouchered at the South Australia State Herbarium (AD), and species identification was verified by Herbarium botanists. All plant specimens were sampled for DNA and sent to Intertek, South Australia (www.intertek.com), for DNA extraction and quantification. Extracts were then normalized to 2 ng/µl in a volume of 100 µl.

#### 2.1.2 Library preparation

The DNA extracts were first sheared to a size distribution peaking around 400–600 bp using a sonicator (Diagenode Bioruptor Pico) run cycle of 15 s On, 90 s Off, and repeat 5 times. Libraries were then generated on the normalized, sonicated DNA extracts, using the NEBNext Ultra II Library preparation kit (New England Biolabs^®^). Manufacturer's instructions were followed with the following modifications: Reactions were done in ^1^/_3_ of the recommended volumes; custom‐made stubby (incomplete, P5 and P7 indexes missing) Y‐adaptors (25 µM) (Glenn et al., 2019) were used at the ligation step. The design of these adapters replaced the uracil excision in the Ultra II protocol as instead, DNA underwent end repair then A‐tailing prior to ligating Y‐adapters. Each adapter had a unique eight nucleotide barcode, giving each sample a unique pair of identical internal molecular identifiers (identified as the eight first base calls for each read). Following adapter ligation, libraries were amplified to detectable concentrations using the supplied Q5 Master Mix at the original reaction volume of 50 µl with in‐house primers P7 preCap Long and P5 preCap Long (cycling conditions: [98°C 10 s, 65°C 30 s, and 72°C 30 s] × 17 cycles, 72°C 120 s, and 4°C hold). The partially complete libraries were then visually checked (2 µL) using gel electrophoresis (1 × TE buffer, 1.5% agarose gel for 40 min at 80 V). The indexed libraries were then pooled according to concentration estimates (determined via visual inspection) into batches of 16 samples and then purified using AMPure XP (at 0.8 × volume concentration) to remove small fragments, remaining oligos, and other impurities.

#### 2.1.3 Multi‐gene bait capture

##### Bait design

We used the RefSeq release of plastid sequences (https://ftp.ncbi.nlm.nih.gov/refseq/release/plastid/ accessed October 2017) to design probes targeting a set of chloroplast gene regions for angiosperms (Appendix S1: Table A2). Using *Arabidopsis lyrata* (GenBank reference NC_034379) as a reference, target regions were extracted from the RefSeq data using Blast (blastn, *e* value <1e−50) and were clustered using CD‐HIT (Li & Godzik, 2006) with a 95% identity cutoff, retaining the longest sequence per cluster for probe design. A total of *c*. 2800 representative sequences, ranging in length from 180 to 900 bp (mean 370 bp), were used to design *c*. 15,000 120‐mer probe sequences with 2X tiling (i.e., each probe overlaps half its length). For more information on bait design, see Waycott et al. (2021).

##### Targeted capture

Targeted capture was performed on each batch of libraries following the myBaits^®^ Targeted NGS Manual Version 4.01 as per the manufacturer's instructions. The hybridization temperature/time was 65°C for 24 h. Following hybridization, the product was amplified using custom P7 and P5 indexed primers designed in‐house using cycling conditions: 98°C 120 s, [98°C 20 s, 60°C 30 s, 72°C 45 s] × 17 cycles, 72°C 30 s, and 4°C hold. The final product was an Illumina library where each sample had a unique combination of identical internal dual barcodes (incorporated during library preparation) and two indexes (incorporated by PCR after hybridization). Within our laboratory, all dual barcode–Index 1–Index 2 combinations are only used once, thus reducing contamination risk.

Following targeted capture and amplification, the resulting libraries were run on a 2100 Bioanalyzer (Agilent) using the high sensitivity DNA assay and molarity was calculated between 300 and 800 bp. All libraries were then pooled in equimolar concentration and purified using AMPure XP (New England Biolabs) at 0.8 × concentration to remove primer dimer and short sequences. The final library underwent further size selection using a Pippin Prep (Sage Science) with a 1.5% agarose gel cassette set to select between 300 and 600 bp. The resulting library was further quantified using an Agilent High Sensitivity D1000 ScreenTape (Agilent) and sent to the Garvan Institute of Medical Research (Sydney, Australia) to be sequenced on one lane of an Illumina HiSeq X Ten using 2 × 150 chemistry.

### 2.2 Bioinformatic analysis

Sequences were demultiplexed based on the P7 index using Illumina Bcl2fastq v2.18.0. The output Read 1 and Read 2 fastq.gz files were then demultiplexed based on the Y‐adapter internal barcodes using AdapterRemoval v2 (Schubert et al., 2016). The following analysis is available in Appendix S2; collapsed and truncated reads were recovered from the AdapterRemoval output and mapped to a reference using BWA‐MEM (Li, 2011). This mapper was chosen as it has consistently been shown to be the most accurate for mapping next‐generation sequencing (NGS) reads of plants (Schilbert et al., 2020; Wu et al., 2019; Yao et al., 2020). The choice of a reference sequence to map each sample to was based on a National Centre for Biotechnology Information (NCBI Resource Coordinators, 2018) search for the closest taxonomic relative, starting from species‐level relation and working up the taxonomic rank until a mutual level was found (Appendix S1: Table A1). SAMtools markdup (Li, 2011) was used to remove PCR duplicates post‐mapping, and variants were called using SAMtools mpileup (Li, 2011) specifying ploidy as 1 and filtering for base quality and mapping quality <30. SAMtools mpileup was chosen as the variant calling tool based on results from variant calling tests using plant NGS data (Schilbert et al., 2020; Wu et al., 2019; Yao et al., 2020). Variant calls were normalized with BCFtools norm (Li, 2011), and BEDtools genomecov (Quinlan & Hall, 2010) was used to create a BED file to replace read coverage (sequence depth) <50 with ambiguous nucleotides (Ns). BCFtools consensus caller was then used to call the consensus FASTA files. These were then imported into Geneious (Geneious Prime^®^ 2020.2.3) and annotated (similarity 25% and 100 bp either side of the gene region) using the closest relative chloroplast reference genome collected from the National Centre for Biotechnology Information (NCBI Resource Coordinators, 2018).

### 2.3 Testing of the reference database

To test the discriminatory ability of the reference database, we conducted a similar analysis to Jones et al. (2021), employing the use of the BLAST (Altschul et al., 1990) to search for sequence similarity in the dataset as is commonly done in metabarcoding studies (Deiner et al., 2017). Each sample was individually searched against the rest of the reference database using BLAST but minus the sample to prevent biasing results. This was done separately for each gene region using rBlast (https://github.com/mhahsler/rBLAST) on R (R core team, 2018), selecting blastn, and only retaining the top hit. Each hit was then classified at the species, genus, family, order, or class level and summarized for each sample.

### 2.4 Choosing a chloroplast barcode

#### 2.4.1 Separate chloroplast gene regions

Utilizing the availability of 20 chloroplast gene regions across 93 temperate coastal plant species, we investigated whether discrimination between taxa improved depending on which chloroplast gene region was used and compared this to using all 20 gene regions. Firstly, each of the 20 target chloroplast gene regions was separately aligned for each specimen in the database using MAFFT (Katoh et al., 2002) with parameters –auto. R (R Core Team, 2018) was then used to compute K2P distances for each alignment using dist.dna and inserting gaps for missing data (Paradis & Schliep, 2019). The sample “*Avicennia marina* St. Kilda” was chosen as the sample to which all other sample distances were measured as all 20 target gene regions were recovered for this sample. In addition, K2P distances were also computed when all available gene regions for each sample were concatenated and aligned, and this was done in R using the seqinr package (Charif & Lobry, 2020) and is denoted as “all.” Plotting these results also included a dendrogram, which was constructed in R with ggdendro (de Vries & Ripley, 2022) using the distances calculated in “all.”

#### 2.4.2 Iterative addition of chloroplast gene regions

For ease of analysis and a prior understanding of the taxonomy of these groups, we separated our reference database into two broad taxonomic (evolutionary) groups (seagrass and saltmarsh/samphire) and conducted K2P distance comparisons (Kimura, 1980) between different levels of relatedness. For the seagrasses, comparisons were conducted using *Amphibolis griffithii* Western Australia Rottnest Island (Cymodoceaceae) as the baseline species, and thus, comparisons included the following: between family (7 samples from Hydrocharitaceae, Zosteraceae), within family (6 samples from the Cymodoceaceae complex; Ruppiaceae, Posidoniaceae), within genus (2 samples of *Amphibolis antarctica*), and within species (2 samples of *Amphibolis griffithii*, separate populations). For the saltmarsh group, all comparisons were determined from *Salicornia quinqueflora* St Kilda. As all species were from Chenopodiaceae, we separated comparisons into groups: Group 1—2 samples of *Chenopodium glaucum*; Group 2—2 samples of *Suaeda australis*; and Group 3—7 species of *Tecticornia*, within genus (2 samples of *Salicornia blackiana*) and within species (1 sample of *Salicornia quinqueflora*, separate populations). The 20 target chloroplast gene regions were ordered by the more commonly used barcoding loci according to those outlined in Hollingsworth et al. (2011), and thereafter ordered randomly. For each comparison (seagrass and saltmarsh/samphire), sequences were iteratively concatenated in R using the seqinr package (Charif & Lobry, 2020) based on the gene order. These were then separately aligned using MAFFT (Katoh et al., 2002) with parameters –auto. R (R Core Team, 2018) was then used to compute K2P distances for each alignment using dist.dna and inserting gaps for missing data (Paradis & Schliep, 2019).

## 3 RESULTS

### 3.1 Reference library generation

**FIGURE 1:**
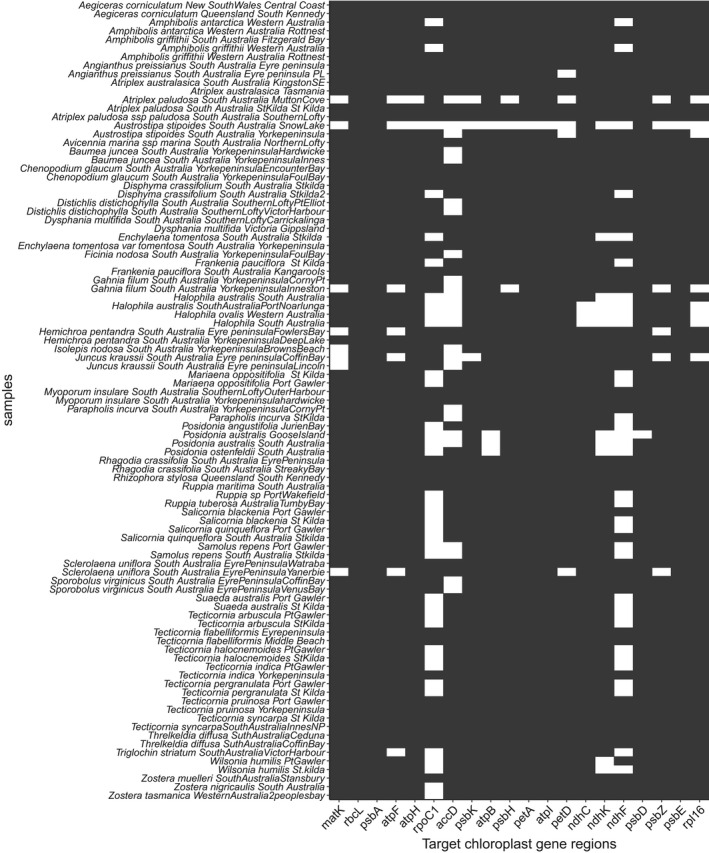
Summary of the 93 coastal plant references generated in this study. Gene recovery is indicated by a colored rectangle, and genes that were not recovered are left blank. Target genes are ordered by the more commonly used plant barcodes on the x‐axis with species and location on the y‐axis

Reference sequences for 93 coastal plant species were generated across 20 target chloroplast gene regions (Figure 1). The maximum number of target gene regions recovered was 20, and the minimum was 4, with an average recovery of 18 chloroplast gene regions across all samples in the database.

### 3.2 Testing of the reference database

**FIGURE 2:**
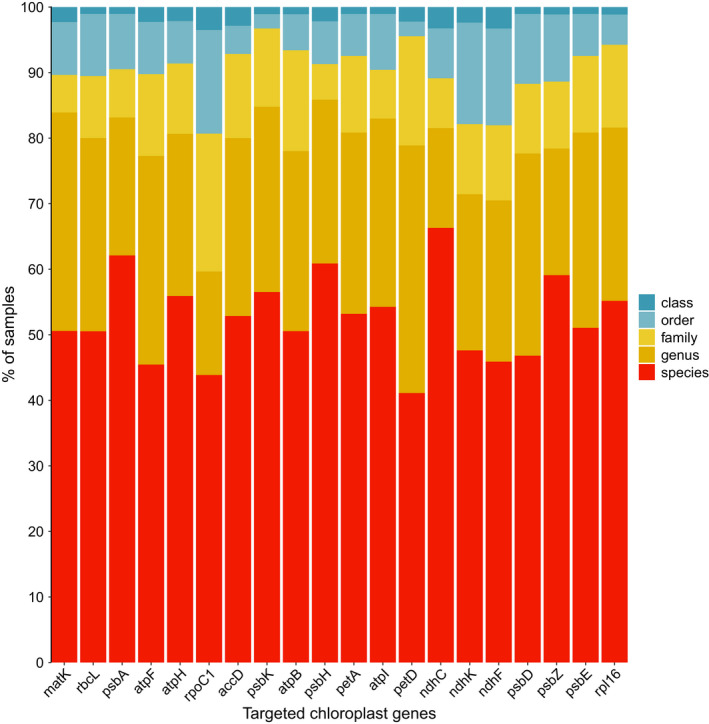
Percentage of samples within the constructed coastal temperate reference database identified at each taxonomic level using BLAST

The utility of the constructed reference database to detect unknown sequences showed variation across the 20 target chloroplast gene regions (Figure 2). The gene region *ndhC* returned the highest number of species‐level matches with 66% of samples matching to species level. Other gene regions *psbA*, *psbH*, and *psbZ* also had high percentage of species‐level matches (62%, 61%, and 59%, respectively). *rpoC1* was the worst‐performing gene region only detecting 44% of samples at species level, and the most classifications at order and class levels of any gene region (16% and 4%, respectively). Overall, all gene regions achieved over 41% species‐level matches (the lowest being 41% for petD), with genus‐level matches ranging from 15 to 38%, family from 5 to 21%, order from 2 to 16%, and class from 1 to 4% of total samples. Overall, 80% of all samples in the reference database could be matched to a sequence at family level or below across all 20 gene regions.

### 3.3 Choosing a chloroplast barcode

**FIGURE 3:**
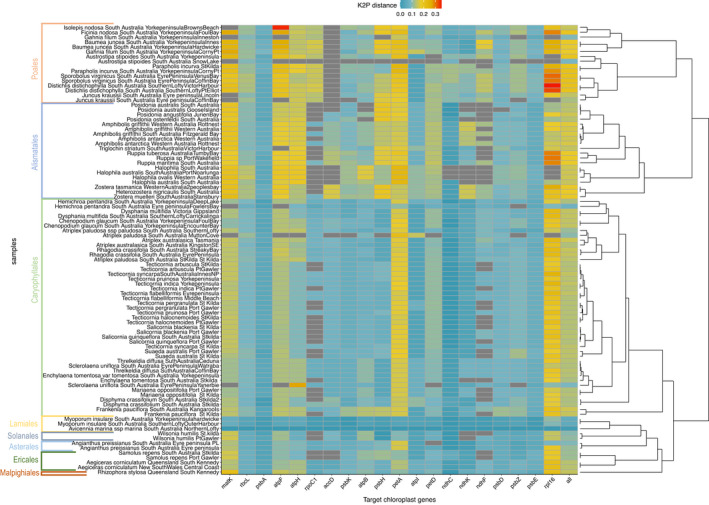
K2P distance measures compared from the sample “*Avicennia marina* St. Kilda” to all other samples within the generated reference database. Colors indicate K2P distance, and samples are highlighted by order on the left. The dendrogram on the right was constructed using K2P distance for all gene regions available for each sample

Comparing genetic distances between samples in the reference database for each of the 20 chloroplast gene regions highlighted that no one gene region confers the same level of discrimination across all samples. For the 20 chloroplast genes used in this study, *rpl16* displayed the largest genetic distance across all comparisons among taxa (Figure 3). Other gene regions that had high genetic distances across the different taxa were *matK*, *petA*, and *atpF*. Specific gene regions conferred greater genetic distance within some orders than others; for example, *psbH* showed higher genetic distance within Alismatales, *atpH* worked better for Alismatales and Poales, and *rpoC1* performed well for Poales (although this gene region was overall poorly recovered across taxa). Using all the available gene regions was shown to generate genetic distances between taxa comparable with *matK*.

**FIGURE 4:**
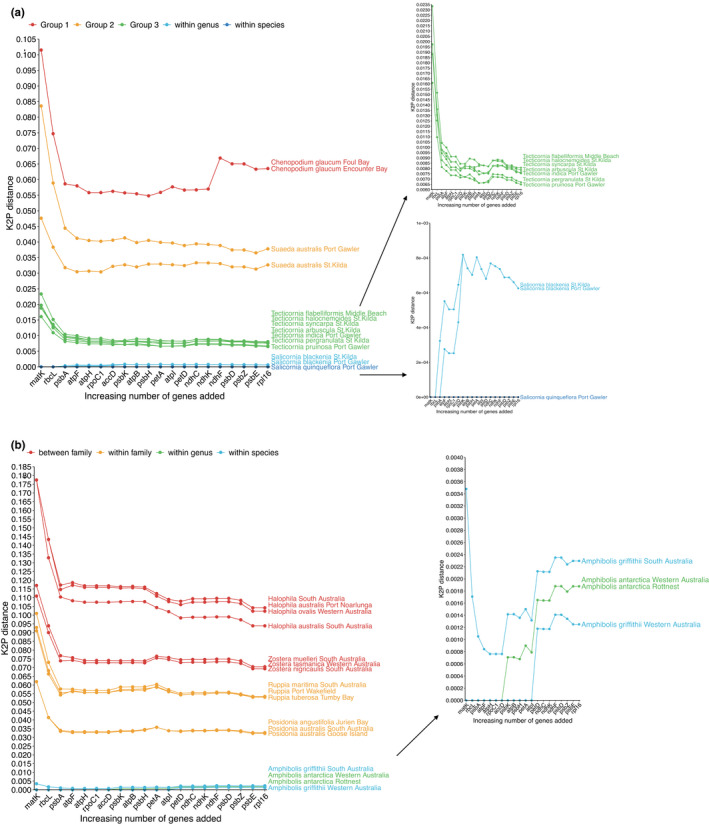
K2P distance comparisons for the chosen saltmarsh (a) and seagrass (b) groups. The sample *Salicornia quinqueflora* St. Kilda was used as the baseline sample to which all other comparisons were made in a. and *Amphibolis grifithii* Rottnest Island for b. Colors indicate the types of comparisons being tested, and close‐up graphs are constructed for species and genus changes to better visualize smaller changes in K2P distance

Greater genetic separation at the species or genus level across all taxa was shown to require additional genes to *matK*, and these were not consistent across the different taxa (Figure 4). For example, the greatest separation of species within *Tecticornia* occurred after the addition of all 20 gene regions (Figure 4a), whereas for *Salicornia*, separation between species and populations occurred with the addition of *atpF*, remained the same when *atpH* and *rpoC1* were added, increased again with the addition of *accD*, but then, genetic distance did not change between taxa and began decreasing for *psbK* and beyond. For the seagrass group comparisons, after all 20 target genes were used, *Halophila australis* had decreased in K2P distance relative to the other *Halophila* species (Figure 4b). Within *Ruppia*, however, the greatest difference in K2P distance between species occurred at *matK*, and by 20 gene regions, this distance had decreased. Finally, for the *Amphibolis* genera, differences in K2P distances for the within‐genus and within‐species comparisons were greatest when the *ndhF* and *psbD* gene regions were used.

## 4 DISCUSSION

This study has demonstrated that targeted capture can be applied to generate multispecies reference libraries for 20 chloroplast gene regions in a single assay. The coastal temperate reference database developed in this study contained 93 plant species across multiple chloroplast gene regions. Exploring the utility of this database to accurately identify unknown sequences highlighted over 80% classification to family level across all genes, but greater taxonomic resolution differed between gene regions. Therefore, the database developed in this study may not be comprehensive enough for unknown sequence assignment at the species or genus level but is adequate at the family level. In addition, we explored differences in K2P distances across the 20 target chloroplast gene regions both separately and using the iterative addition of gene regions. Our findings highlight that different gene regions yield varying abilities to separate taxa across divergent plant groups. Overall, this highlights a multigene region approach to generating references is necessary for consistent taxonomic discrimination across many plant groups.

### 4.1 Generating a reference database using targeted capture

A targeted capture approach to reference sequence generation means we can generate references across multiple plant taxa and gene regions in a single assay, much more efficiently than standard (PCR‐based) DNA barcoding. This increases our ability to generated barcodes for a variety of flowering plant taxa for decreased effort and an increased number of barcodes per species. In addition, this approach does not rely on initial PCR amplification of a targeted gene region, thus overcoming biases induced by PCR (Coissac et al., 2012). This study has shown that up to 16 samples can be pooled per targeted capture reaction and post‐capture libraries can be pooled for 384–480 samples (~4–5 plates; Waycott et al., 2021) for sequencing (Illumina Novaseq). This is a substantial number of samples that can be processed for reference generation in a single sequencing effort, and this study has shown this can occur for up to 20 chloroplast gene regions. The average recovery of target chloroplast gene regions for samples in our database was 92% across all reference samples, noting a substantially lower gene recovery for the samples “*Atriplex paludosa South Australia MuttonCove*” and “*Austrostipa stipoides South Australia SnowLake*,” which is likely due to insufficient or reduced quality of DNA extract for these samples. Replicates for these species from different locations recovered 20 and 17 genes, supporting the conclusion that it is likely to be an issue‐specific to the plant material for these samples. Fortunately, for samples that are suspected to be of low quality or unable to yield high concentrations of DNA, this protocol can be altered by reducing pooling during capture and sequencing, or by increasing hybridization time.

### 4.2 Testing the generated reference database

Testing the utility of this reference database for unknown species assignment using BLAST highlighted that it may not be comprehensive enough for genus‐ and species‐level assignment but is adequate for family‐level assignment. However, given the fact reference databases are depauperate for Southern Hemisphere species, particularly coastal plants, and are mostly limited to a few gene regions (i.e., Barcode of Life Database; *matK* and *rbcL*), this database is a significant step toward generating comprehensive reference databases for this region. Furthermore, classification of unknowns will improve with the addition of more taxa and the approach we suggest in this study will increase the efficiency of generating these references. For metabarcoding studies, having the ability to conduct sequence matching to 20 genes instead of just one means we have a greater chance of finding a match at high taxonomic resolution as, evidently, some gene regions performed better than others for sequence assignment at species, genus, and family levels.

### 4.3 Which chloroplast gene to use?

The 20 chloroplast gene regions used in this study confer different genetic distances between taxa, which is highly important in deciding which region to use as a plant barcode. The gene regions *matK*, *rpl16*, and *atpF* appeared to offer the greatest discrimination between samples across all orders, with other regions performing better for some taxa and not others (e.g., *rpoC1*, *psbH*, and *petA)*. Moreover, we showed that the addition of all 20 chloroplast gene regions does not necessarily confer greater genetic distance estimates, which is presumably due to an increasing number of invariant characters in the matrix as gene regions are added (e.g., *ndhC*, *psbE*), leading to, on average, less differences. We further investigated whether the 20 chloroplast gene regions in this study performed better for species separation when multiple regions were used iteratively. The addition of chloroplast regions beyond *matK* decreased K2P distance for all comparisons in Figure 4, although this then reached a plateau after three gene regions. However, we did notice the addition of chloroplast gene regions increased K2P distance at the genus and species level. This may mean the genetic information required to separate families, and groups within families, is contained within the *matK* gene region, but species‐level changes require additional gene regions. Species within the *Amphibolis*, *Tecticornia*, and *Salicornia* genera all showed increases in K2P distance between taxa as the number of chloroplast gene regions increased, but this effect was variable among the included gene regions. Therefore, we confirm there is no one‐size‐fits‐all approach to plant barcodes (Kress et al., 2005); rather, we highlight that multi‐gene methods are necessary for distance‐based approaches across multiple taxon groups.

As this work has focused specifically on generating references for chloroplast gene regions, it has not included the commonly used barcode, the nuclear ribosomal internal transcribed spacer (ITS) region. This gene region is likely to offer improved discrimination among samples and has been proposed as a standard plant barcode (Banchi et al., 2020). Inclusion of nuclear regions would be possible using nuclear baits (Johnson et al., 2019; Waycott et al., 2021) as this approach has also been found to recover ITS as by‐catch (Nge et al., 2021). However, it should be noted that inclusion of nuclear regions would come with additional analytical issues such as paralogy and ploidy. Overcoming these analytical challenges, however, will further enhance species identification as chloroplast gene regions are not capable of disentangling hybridization that occurs—which is a limitation of generating references for only chloroplast gene regions.

## 5 CONCLUSIONS

Reference sequence databases are critical for genomic projects. The lack of reliable reference sequence databases for a wide range of taxa, and an efficient method to generate them, is stifling the development, application, and correct interpretation of metabarcoding research. This study has shown that some of these limitations may be overcome by using a targeted capture approach, in combination with a specially designed bait set to capture multiple chloroplast gene regions across all flowering plant communities in a single assay. This study successfully generated a reference sequence database for 20 chloroplast gene regions across 93 plant specimens using targeted capture and could identify unknown sequences to family level for 80% of samples, with the ability for this to improve with the addition of more taxa. Further, findings of this work have highlighted that the different gene regions used in this study confer varying levels of discrimination among taxa. For greater taxonomic resolution, additional gene regions need to be used other than the standard plant barcodes (*matK*, *rbcL*) and this will require more effort as reference databases will need to be built. Ultimately, no single chloroplast barcode works well across all plant groups, highlighting the need for reference generation across multiple gene regions and this study has shown targeted capture can achieve this. Applying this method and designing additional bait sets mean plant references can be generated beyond just flowering plants but to additional plant groups to achieve reference DNA sequence databases for the world's plants.

## CONFLICT OF INTEREST

The authors declare no conflicts of interest.

## AUTHOR CONTRIBUTIONS

**Nicole Foster:** Conceptualization (lead); Data curation (lead); Formal analysis (lead); Funding acquisition (supporting); Investigation (lead); Methodology (equal); Project administration (equal); Software (equal); Writing – original draft (lead); Writing – review & editing (lead). **Kor‐jent Van Dijk:** Conceptualization (equal); Data curation (equal); Methodology (equal); Supervision (equal). **Edward Biffin:** Conceptualization (equal); Data curation (equal); Formal analysis (supporting); Methodology (equal); Supervision (equal); Writing – review & editing (supporting). **Jennifer Young:** Writing – review & editing (equal). **Vicki Thomson:** Formal analysis (supporting); Software (equal); Writing – review & editing (equal). **Bronwyn Gillanders:** Supervision (equal); Writing – review & editing (equal). **Alice R. Jones:** Funding acquisition (lead); Project administration (equal); Supervision (equal); Writing – original draft (equal); Writing – review & editing (equal). **Michelle Waycott:** Conceptualization (lead); Formal analysis (supporting); Funding acquisition (equal); Investigation (equal); Methodology (supporting); Project administration (supporting); Supervision (lead); Writing – original draft (supporting); Writing – review & editing (equal).

## Supporting information

Appendix S1‐S2

## Data Availability

DNA sequences are deposited on GenBank accessions SAMN20368835‐SAMN20368927; NCBI SRA; and TLS: PRJNA749385.
